# CellOntologyMapper: Consensus mapping of cell type annotation

**DOI:** 10.1002/imo2.70064

**Published:** 2025-11-06

**Authors:** Zehua Zeng, Xuehai Wang, Hongwu Du, Cencan Xing

**Affiliations:** ^1^ School of Chemistry and Biological Engineering University of Science and Technology Beijing Beijing China; ^2^ Daxing Research Institute University of Science and Technology Beijing Beijing China; ^3^ Department of Genetics Stanford University Stanford California USA; ^4^ Karolinska Institute, Department of Learning, Informatics, Management and Ethics Tomtebodavägen Solna Sweden; ^5^ Department of Maternal, Child and Adolescent Health School of Public Health Anhui Medical University Hefei China

## Abstract

CellOntologyMapper automates the standardization of cell type annotations in single‐cell RNA‐seq by mapping user‐defined names to the Cell Ontology (CL) and Cell Taxonomy (CT). The framework constructs a comprehensive embedding database of 19,381 curated cell types using sentence transformers, large language models, and synonym expansion. User inputs, such as abbreviations (e.g., “dNK”), are processed through embedding similarity ranking and LLM amplification to resolve ambiguous or context‐dependent labels. The system outputs standardized ontology terms with high accuracy (0.835 for CL, 0.911 for CT). Implemented as a Python package within the OmicVerse ecosystem and available via a web interface, CellOntologyMapper enables reproducible, large‐scale single‐cell meta‐analysis by unifying diverse nomenclatures across studies, tissues, species, and disease states.

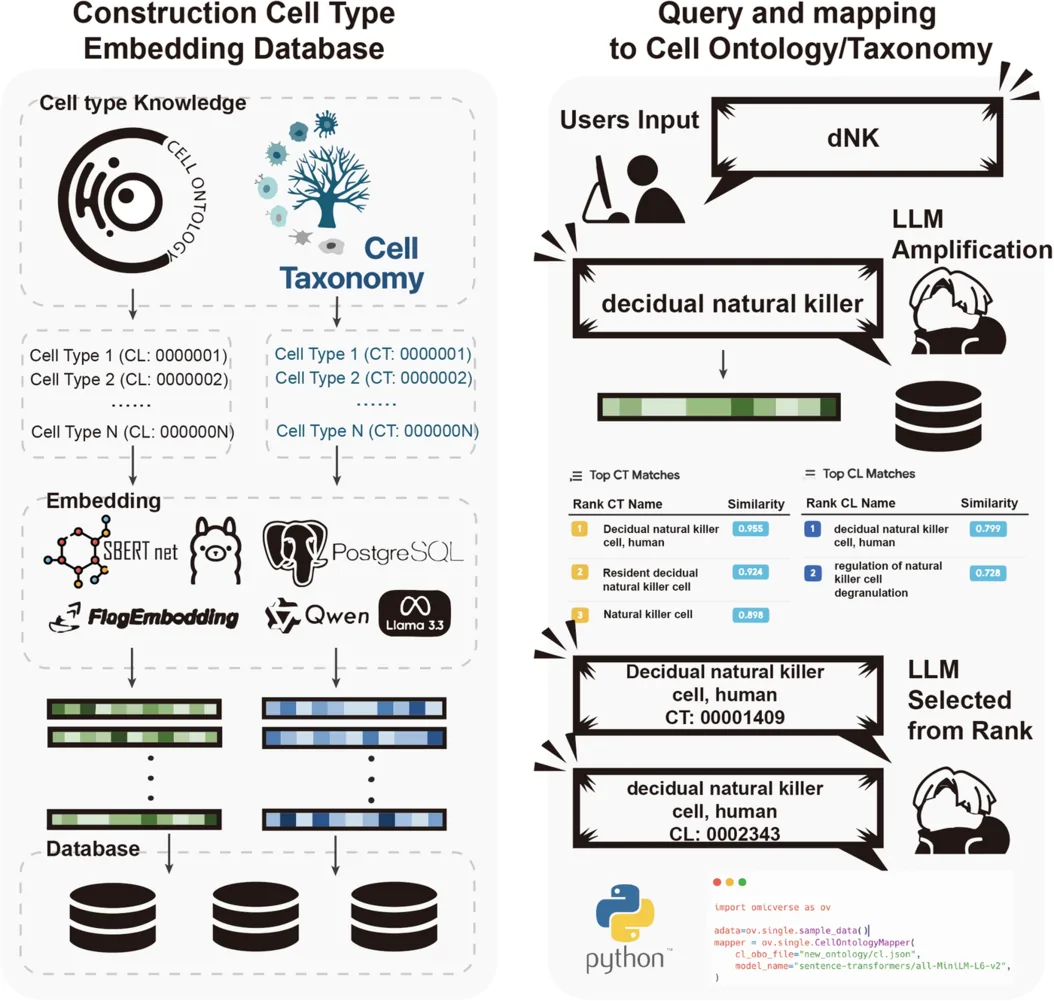

## AUTHOR CONTRIBUTIONS

**Zehua Zeng**: Conceptualization; methodology; software; data curation; investigation; validation; writing—original draft; formal analysis; visualization. **Xuehai Wang**: methodology; software; data curation; investigation; **Hongwu Du**: Conceptualization; Writing—review and editing. **Cencan Xing**: Artificial expert annotation of cell types, revised manuscript. All authors have read the final manuscript and approved it for publication.

## CONFLICT OF INTEREST STATEMENT

The authors declare no conflicts of interest.

## ETHICS STATEMENT

No animals or humans were involved in this study.

To the Editor,

Single‐cell RNA sequencing has revolutionized our understanding of cellular diversity across tissues, developmental stages, and disease states. As single‐cell atlases continue to expand—with some now encompassing over 100 million cells—accurate cell type annotation has emerged as a fundamental prerequisite for meaningful biological interpretation and cross‐study integration [1].

Despite this critical importance, the single‐cell genomics field faces a persistent challenge: researchers employ vastly different naming conventions when annotating cell types, creating a fragmented landscape that impedes data integration and comparative analysis [2]. While individual studies may use descriptive names like “NEUT,” “dM” or simply “Mac1,” these labels often refer to overlapping or identical cell populations, making cross‐data set comparisons extraordinarily difficult [3]. This challenge becomes even more complex in cross‐species studies, where equivalent cell types may be annotated with species‐specific nomenclatures that obscure evolutionary relationships and functional conservation patterns [4]. Current approaches to address this challenge—re‐annotating integrated datasets or manual harmonization—introduce substantial limitations [5]. Re‐annotation after data integration risks batch effect confounding, while manual harmonization is labor‐intensive, subjective, and scales poorly with expanding data volumes [6].

The scientific community has developed standardized ontologies, notably the Cell Ontology (CL) and Cell Taxonomy (CT) databases, which provide hierarchical, controlled vocabularies for cell type classification [7]. However, adoption remains inconsistent across the research community, with many studies continuing to use laboratory‐specific nomenclatures [8].

To bridge this critical gap, we developed CellOntologyMapper (https://github.com/Starlitnightly/CellOntologyMapper), a computational framework that automatically standardizes cell type annotations by mapping user‐defined cell names to established CL [9] and CT [10] identifiers. Our approach leverages state‐of‐the‐art natural language processing techniques, including advanced sentence transformers and large language models (LLMs), to intelligently interpret diverse naming conventions and resolve them to standardized ontological terms. The system handles common challenges, including abbreviated cell names, synonym resolution, and context‐dependent interpretation based on tissue origin and experimental conditions [7]. The framework is implemented as an accessible Python package within the OmicVerse [11] ecosystem, designed to integrate seamlessly into existing single‐cell analysis workflows. Besides, users can also use our online website to perform the cell type name mapping (https://omicverse.com/cellmapper/).

## 1 OVERVIEW OF CELLONTOLOGYMAPPER DESIGN

To address the challenge of inconsistent cell type naming across single‐cell studies, we developed CellOntologyMapper, a comprehensive framework that standardizes cell type nomenclature through two interconnected and complementary modules (Figure 1A,B). The first module is dedicated to constructing an extensive Cell Ontology Query Database, which serves as a foundational resource for accurate cell type mapping. To achieve this, we meticulously extracted cell type names from authoritative sources, including the well‐established CL and CT databases. We then employed state‐of‐the‐art sentence transformer models, including all‐MiniLM‐L6‐v2, FlagEmbedding (BAAI), and Qwen (Alibaba), to generate high‐dimensional embedding vectors that capture semantic relationships between cell type names [12, 13]. These embeddings, linked to unique cell type identifiers, were systematically organized within an optimized SQL database infrastructure, enabling rapid and accurate similarity searches (Figure 1A).

**FIGURE 1:**
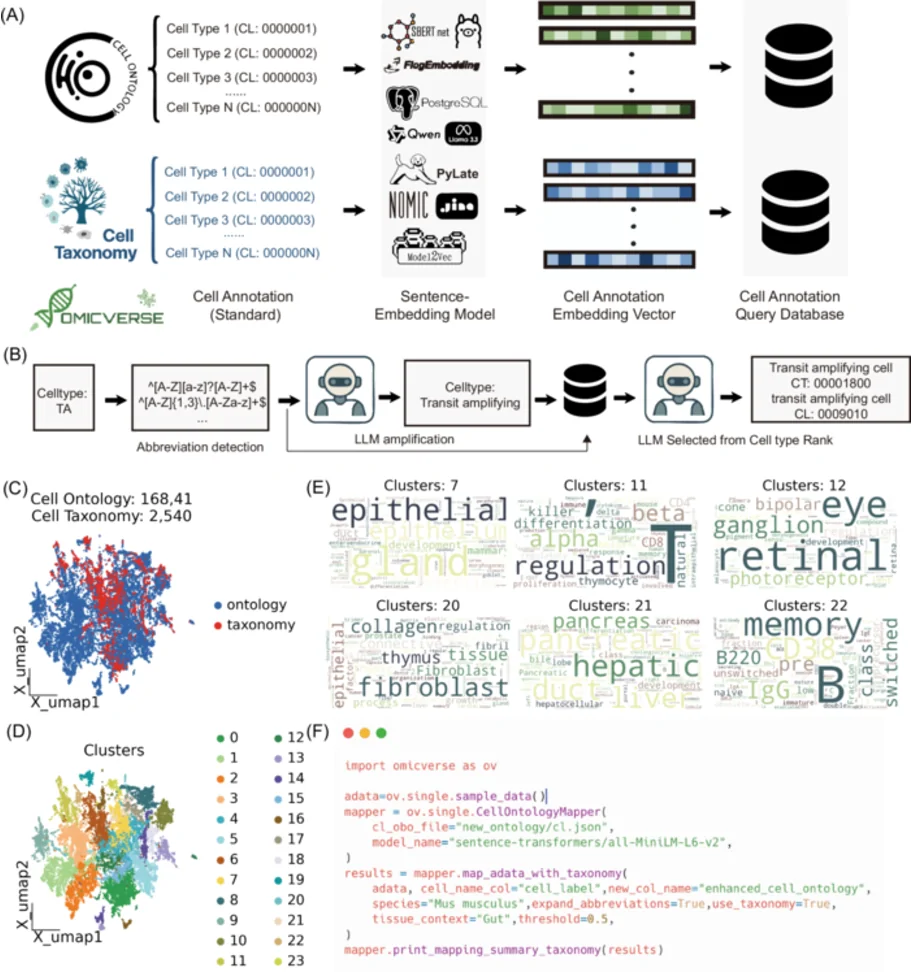
Overview of CellOntologyMapper for Comprehensive Cell Type Standardization. (A) Workflow of constructing the Cell Ontology Query Database. Cell type names were extracted from Cell Ontology and Cell Taxonomy databases, embedding vectors were generated using state‐of‐the‐art sentence transformers (FlagEmbedding, Qwen), and subsequently stored in an SQL database for efficient querying. (B) Pipeline for mapping user‐provided cell type abbreviations (e.g., “TA”) to standardized names using regular expression (regex) detection followed by Large Language Model (LLM) amplification. The LLM suggests full cell type names based on sequencing context and tissue background, computes their embedding vectors, ranks similarities against database entries, and selects appropriate Cell Ontology (CL) and Cell Taxonomy (CT) identifiers from top candidates. (C) Summary of the constructed query database containing 19,381 cell type entries (16,841 from Cell Ontology, 2540 from Cell Taxonomy), visualized on a UMAP embedding to illustrate the distribution of database entries. (D) Unsupervised clustering of cell type embeddings from the query database. Principal component analysis (PCA), neighborhood graph construction, and Leiden clustering identified 24 distinct clusters, each color‐coded. (E) Word cloud visualizations highlighting representative cell types from selected clusters. Cluster 7 primarily includes epithelial‐related cell types; Cluster 11 is enriched in T cells and subtypes; Cluster 12 consists predominantly of retinal‐related cells; Cluster 20 represents fibroblast‐related entries; Cluster 21 groups cells associated with liver and pancreas; and Cluster 22 predominantly includes B cells and their subtypes. (F) Example code snippet demonstrating the straightforward and user‐friendly API provided by OmicVerse for efficient cell type annotation mapping.

The second module implements an intelligent, context‐aware mapping pipeline aimed at converting user‐provided cell type terms into standardized ontology terms. A common hurdle in cell type annotation is the widespread use of abbreviated or ambiguous naming conventions. To robustly address this, we apply regular expression algorithms to detect and flag abbreviated terms. Upon identification of these abbreviations, we harness the capabilities of LLMs to generate contextually accurate full‐length cell type names based on experimental context, such as tissue type, developmental stage, and sequencing protocol compute methodology [14, 15]. Subsequently, embedding vectors for these expanded query terms are computed, facilitating their rapid similarity ranking against entries within the comprehensive database. Finally, an LLM‐assisted selection agent identifies the most appropriate CL and CT identifiers from the top‐ranked candidates, ensuring both semantic accuracy and biological relevance (Figure 1B).

Our comprehensive query database encompasses 19,381 curated cell type entries, comprising 16,841 from CL and 2540 from CT (Figure 1C). To investigate the semantic organization of this database, we performed dimensionality reduction using Uniform Manifold Approximation and Projection (UMAP) from the term embedding vector and applied unsupervised Leiden clustering, revealing 24 distinct clusters that group semantically related cell types (Figure 1D). Remarkably, these clusters exhibit clear biological coherence: cluster 7 predominantly contains epithelial cell types, cluster 11 is enriched for T cells and their specialized subtypes, cluster 12 groups retinal cell populations, cluster 20 encompasses fibroblast‐related entries, cluster 21 aggregates hepatic and pancreatic cell types, and cluster 22 primarily consists of B cell lineages and their developmental stages (Figure 1E). This clustering pattern validates the biological meaningfulness of our embedding approach and demonstrates the system's capacity to capture nuanced relationships within the cellular taxonomy.

To ensure broad accessibility, we provide a streamlined, user‐friendly Application Programming Interface (API) through the OmicVerse package in Python that enables researchers to perform cell type standardization with minimal code requirements (Figure 1F). Besides, we also provide an online website to help researcher obtain the accurate cell name: https://omicverse.com/cellmapper/. This implementation facilitates seamless integration into existing single‐cell analysis workflows while maintaining the sophisticated mapping capabilities of the underlying framework.

## 2 CELLONTOLOGYMAPPER DEMONSTRATES ROBUST CROSS‐SCALE PERFORMANCE ACROSS DIVERSE TISSUE CONTEXTS AND ANNOTATION COMPLEXITIES

To rigorously evaluate the versatility, precision, and robustness of CellOntologyMapper, we conducted comprehensive validation using multiple independent single‐cell datasets spanning diverse scales, biological contexts, and annotation complexities. This systematic assessment included small‐scale datasets (<20 cell types, Data from Vieira Braga et al. [16] and Haber et al. [17]), medium‐scale studies (20–50 cell types, Data from Zeng et al. [18]), large‐scale atlases (>50 cell types, Data from Domínguez Conforme et al. [19]), and particularly challenging datasets characterized by extensive abbreviations, ambiguous terminology and intricate developmental lineages (Data from Arutyunyan et al. [20]). We first tested CellOntologyMapper on a lung tissue data set containing 17 well‐characterized cell types, including immune populations, structural cells, and specialized pulmonary cell lineages (Figure 2A, Table S1). And then we tested CellOntologyMapper on Bone marrow included 24 and 55 cell types (Figure S1, Table S1) and cross tissue immune cells included 91 cell types (Figure S2, Table S1). CellOntologyMapper achieved perfect accuracy, precisely mapping each annotated cell type to its corresponding Cell Taxonomy identifier. Notably, it effectively distinguished between closely related cell populations such as alveolar macrophages, interstitial macrophages, and distinct dendritic cell subsets, underscoring its capacity for precise annotation within conventional tissue‐specific contexts.

**FIGURE 2:**
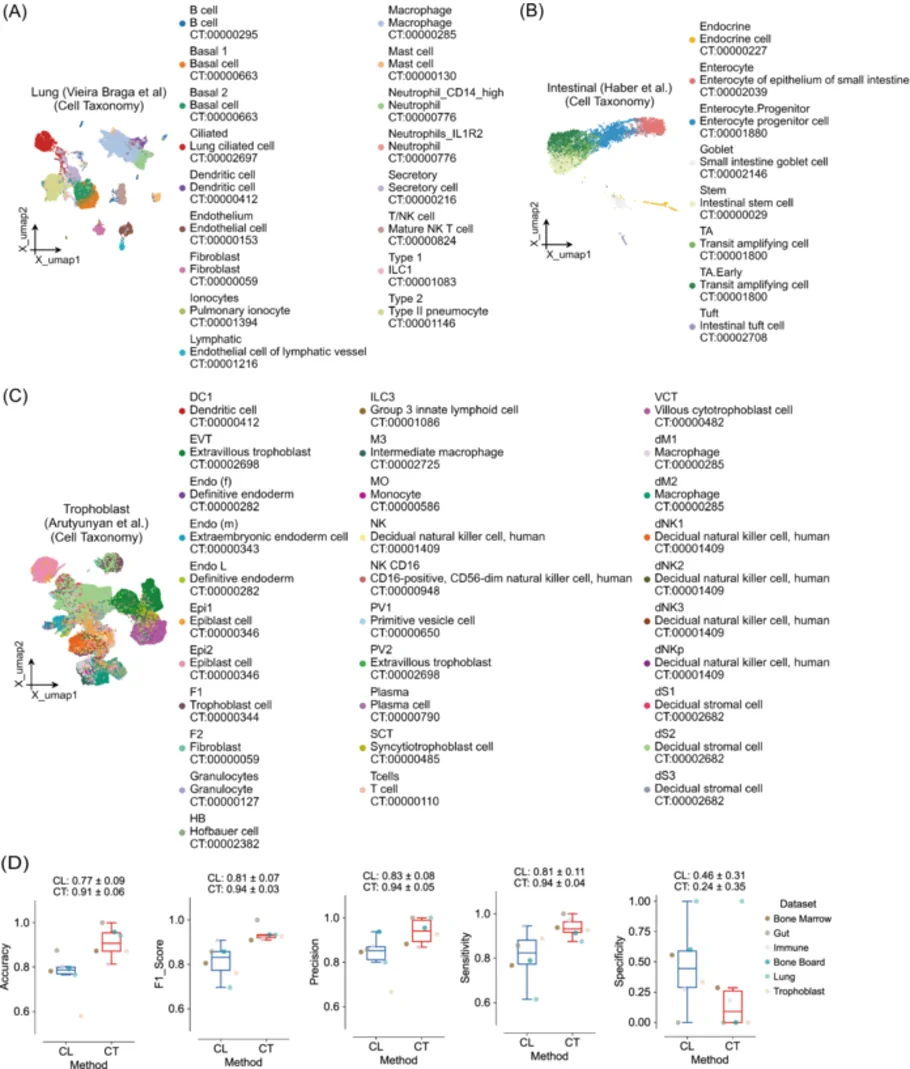
Cell taxonomy‐based annotation of single‐cell datasets across multiple tissues. (A) UMAP visualization of annotated lung single‐cell data set (Vieira Braga et al.) based on Cell Taxonomy. Identified cell types include immune cells (e.g., B cells, macrophages, mast cells), structural cells (basal cells, fibroblasts), and specialized epithelial cells (ciliated, secretory). (B) Annotation of intestinal single‐cell data (Haber et al.) visualized by UMAP, highlighting differentiation stages and specialized epithelial populations such as enterocytes, transit amplifying cells (TA), goblet cells, and endocrine cells. (C) UMAP‐based annotation of trophoblast single‐cell data set (Arutyunyan et al.), demonstrating diverse placental cell populations including extravillous trophoblasts (EVT), decidual stromal cells (dS), natural killer cells (NK), and syncytiotrophoblasts (SCT), annotated comprehensively via Cell Taxonomy (CT) identifiers. (D) Boxplots comparing performance metrics for CL (blue) versus CT (red) mappings across six datasets (Bone Marrow, Gut, Immune, Bone Board, Lung, Trophoblast): Accuracy, F1‑score, Precision, Sensitivity, and Specificity, with mean ± SD indicated above each box. Data points for individual datasets are overlaid as jittered circles, facilitating visualization of data set‑wise variability.

To evaluate the method in contexts of increased biological complexity and annotation ambiguity, we next applied CellOntologyMapper to an intestinal epithelial data set (Haber et al. [17]) characterized by extensive cellular differentiation gradients and rare specialized populations. This data set presented unique annotation challenges, including multiple intermediate differentiation states, region‐specific epithelial variants, and highly specialized cell types such as tuft cells and enteroendocrine subtypes. CellOntologyMapper successfully navigated this complexity, accurately annotating diverse populations from proliferative transit‐amplifying cells to terminally differentiated absorptive enterocytes, while correctly identifying rare populations like transit amplifying and goblet cell subtypes (Figure 2B, Table S1). This performance highlights its ability to handle nuanced biological contexts in which cell identity spans continuous developmental and differentiation spectra.

We subjected CellOntologyMapper to an even more stringent test using a highly complex trophoblast data set from Arutyunyan et al. [20], characterized by intricate placental developmental lineages and frequent utilization of abbreviated and ambiguous nomenclature. This data set incorporates various embryonic cell populations, including syncytiotrophoblasts, cytotrophoblasts, and multiple extravillous trophoblast subtypes, often represented by acronyms and short forms common in developmental biology literature. Despite these substantial challenges, CellOntologyMapper exhibited outstanding performance, successfully resolving ambiguous abbreviations and accurately mapping each annotated population to validated Cell Taxonomy terms. Critically, the system preserved biological coherence across distinct developmental trajectories, effectively differentiating closely related progenitor and differentiated trophoblast states, demonstrating sophisticated semantic understanding and contextual annotation accuracy (Figure 2C, Table S1).

To further substantiate the versatility and scalability of CellOntologyMapper, we then evaluate the accuracy, F1‐score, precision, sensitivity, and specificity. Specifically, aggregated results from these datasets revealed that CL‐based mapping achieved a mean accuracy of 0.835 ± 0.036, precision of 0.894 ± 0.058, recall of 0.793 ± 0.069, F1 score of 0.837 ± 0.033, sensitivity of 0.793 ± 0.069, and specificity of 0.460 ± 0.341. Moreover, CT‐based mapping demonstrated even higher performance, achieving a mean accuracy of 0.911 ± 0.055, precision of 0.933 ± 0.061, recall of 0.930 ± 0.049, F1 score of 0.931 ± 0.051, sensitivity of 0.930 ± 0.049, and specificity of 0.245 ± 0.389 (Figure 2D).

CellOntologyMapper represents the first comprehensive, automated solution for systematically standardizing cell type nomenclature through intelligent mapping to established ontological databases. Through extensive validation across a broad spectrum of biological contexts, our framework demonstrated robust performance consistently, irrespective of data set scale or annotation complexity. The significance of CellOntologyMapper transcends mere technical enhancement, fundamentally enabling transformative advances in single‐cell biology by addressing longstanding annotation fragmentation challenges.

By facilitating uniform annotation practices, CellOntologyMapper empowers researchers to perform robust, large‐scale meta‐analyses, unlocking novel insights from integrative analyses across multiple studies. This capability is critical as the single‐cell biology community collectively endeavors to build comprehensive cellular atlases that span diverse species, developmental stages, and disease states. Notably, our tool exhibits exceptional proficiency in handling abbreviated nomenclatures, ambiguous terminology, and context‐specific annotation practices, ensuring widespread compatibility across diverse research communities.

Integration of CellOntologyMapper within the OmicVerse ecosystem, offering both a Python‐based API (https://omicverse.readthedocs.io/en/latest/Tutorials‐single/t_cellmatch/) and an intuitive online interface (https://omicverse.com/cellmapper/) for easy access (also see the Section Procedure in the supplement) and use, provides an accessible, intuitive, and streamlined solution for annotation standardization, promoting rapid adoption within the single‐cell research community. Researchers incorporating CellOntologyMapper‐derived annotations in their publications enhance the credibility, broader applicability, and scientific utility of their cell type annotations. As single‐cell datasets and atlases continue to grow exponentially in scale and complexity, automated and standardized annotation will become indispensable for maintaining rigorous scientific standards and enabling meaningful cross‐study comparisons. Ultimately, CellOntologyMapper represents a pivotal advancement toward realizing a fully integrated single‐cell genomics ecosystem, maximizing the collective scientific value derived from ongoing cellular discoveries.

## Supporting information

Supplementary 0128 v4.

**Table S1.** Celltype mapping result using CellOntologyMapper.

## Data Availability

The data and scripts used are saved in GitHub (https://github.com/Starlitnightly/CellOntologyMapper). Supplementary materials (methods, figures, tables, graphical abstract, slides, videos, Chinese translated version, and update materials) may be found in the online DOI or iMetaOmics Science http://www.imeta.science/imetaomics/.
