# Supplementary material for: CellOntologyMapper: Consensus mapping of cell type annotation
Source: IMetaOmics. 2025 Nov 6;3(1):e70064. doi: 10.1002/imo2.70064 (PMC13674396; doi:10.1002/imo2.70064)
Supplement: Supplementary file 1 [file IMO2-3-e70064-s001.docx]

Supporting information to

CellOntologyMapper: Consensus mapping of cell type annotation

Zehua Zeng^1,2,3#^, Xuehai Wang^4,5#^, Hongwu Du^1,2^*, Cencan Xing^1,2^*

^1^School of Chemistry and Biological Engineering, University of Science and Technology Beijing, Beijing, China

^2^Daxing Research Institute, University of Science and Technology Beijing, Beijing, China

^3^Department of Genetics, Stanford University, CA, USA

^4^Karolinska Institute, Department of Learning, Informatics, Management and Ethics, Tomtebodavägen, Solna

^5^Department of Maternal, Child and Adolescent Health, School of Public Health Anhui Medical University, Hefei, China.

^#^These authors contributed equally: Zehua Zeng, Xuehai Wang

*Correspondence: [hongwudu@ustb.edu.cn](mailto:hongwudu@ustb.edu.cn) (Hongwu Du), [cencanxing@ustb.edu.cn](mailto:cencanxing@ustb.edu.cn) (Cencan Xing)

Material and Methods

## Sample Sources and Data Acquisition

**Bone Marrow Reference Map.** We obtained single‑cell RNA‑sequencing data from Zeng *et al*[1]. (PMID: 40294241), which generated a comprehensive hematopoietic reference atlas in human bone marrow. Two annotation schemes were extracted:

1. **Medium‑scale set 1 (24 cell types):** the AuthorCellType_Board classification, encompassing major hematopoietic lineages and progenitor subsets.
2. **Medium‑scale set 2 (55 cell types):** the full AuthorCellType scheme, resolving finer gradations within progenitors and mature compartments.
    Raw count matrices and metadata were downloaded from the CellxGene portal (collection ID: f6c50495-3361-40ed-a819-fb9644396ed9).

**Intestinal Epithelium.** We accessed the *Haber et al*[2]*.* mouse small‑intestinal scRNA‑seq dataset (PMID: 29144463), which profiles epithelial cells under homeostatic and perturbed conditions. We selected the cell_label annotations defining eight canonical epithelial cell types (e.g., enterocytes, goblet cells) as our “small‑scale” benchmark. Data files were retrieved from Figshare (file ID: 54169301).

**Cross‑Tissue Immune Compendium.** The Domínguez Conforme *et al*[3]*.* resource (PMID: 35549406) provides a large‑scale atlas of innate and adaptive immune cells across multiple human tissues. We utilized the Predicted_labels_CellTypist classification, which assigns 91 immune cell types, to define our “large‑scale” dataset. Count matrices and annotations were downloaded from CellxGene (collection ID: 62ef75e4-cbea-454e-a0ce-998ec40223d3).

**Pulmonary Cellular Landscape.** Data from Vieira Braga *et a*.[4] *l.* (PMID: 31209336) comprise three healthy lung 10X/Drop‑seq transplant replicates and lung‑biopsy samples. Following the authors’ recommendation, nasal and brush‑derived samples were excluded. We extracted the cell_type annotation for 17 major pulmonary cell subsets and obtained the data from Figshare (file ID: 24539942).

**Human Maternal–Fetal Interface.** Arutyunyan *et al.*[5]*.* (PMID: 36991123) characterized the full human maternal–fetal interface, including decidua, chorion, and myometrium. We used the celltype_predictions annotation, comprising 31 trophoblast and stromal identities, to assemble a medium‑scale dataset. Annotation nomenclature is simplified (e.g., “EVT,” “VCT”), presenting mapping challenges. Data were accessed via CellxGene (collection ID: e2c257e7-6f79-487c-b81c-39451cd4ab3c).

## Preprocessing and Visualization

All count matrices were standardized using OmicVerse’s lazy loading API (omicverse.single.lazy) to harmonize data ingestion. We applied scVI[6] (version 1.0) for batch‐effect removal, treating each dataset as an independent batch. After model convergence (early stopping at 50 epochs, learning rate 1e‑3), we extracted the 10-dimensional latent embedding and performed UMAP projection (n_neighbors = 15, min_dist = 0.3) for two‐dimensional visualization of cell‐type annotation consensus across studies.

## Cell Type Name Embedding

To represent cell‐type names in a continuous vector space, we employed the Sentence‑Transformers implementation of the all‑MiniLM‑L6‑v2 model (Reimers & Gurevych, *EMNLP* 2020). We constructed two embedding repositories:

1. **Ontology terms:** Each entry from the Cell Ontology[7] (CL, version 2025.06.01) and Cell Taxonomy[8] (CT, version 2022.07.29) was tokenized and encoded to a 384‑dimensional vector; stored in a MySQL table (ontology_embeddings).
2. **Query names:** For each human‐annotated cell type (from all five datasets), we generated embeddings on‐the‐fly under identical model parameters.

All embeddings were normalized (ℓ₂ norm) to facilitate cosine similarity computations.

## Expansion of Abbreviated Cell Type Names via LLM Agents

To resolve domain‐specific abbreviations (e.g., “dM,” “dNK”), we developed two automated pipelines leveraging a large language model (LLM) backbone (GPT‑4 API):

- **CellOntology‑Agent‑Query:** Accepts an abbreviation and a brief research‐context prompt (“Mapping trophoblast subpopulations in the human maternal–fetal interface”) to generate up to five candidate full‐name expansions (e.g., “decidual Macrophage,” “decidual Natural Killer cell”).
- **CellOntology‑Agent‑Determiner:** Takes the candidate list and evaluates each against the context prompt, returning the single best‐matched full name based on model confidence scores.

All LLM calls were performed with temperature = 0.0 to maximize determinism and logged for reproducibility.

## Cell Type Name Similarity and Mapping

For each query embedding, we computed cosine similarity against all ontology embeddings. The top K = 20 candidates were first retrieved using a Sentence‐Transformer index (Faiss). We then applied a Cross‑Encoder re‐ranking step (scBERT fine‐tuned on cell‐ontology pairs) to refine the top 5 matches. Only candidates with cosine similarity > 0.6 were retained; if none met this threshold, the mapping was set to “Unknown.” Final mapping outputs record both the CL term identifier and preferred label.

## Evaluation of Mapping Accuracy

To benchmark our **CellOntologyMapper** tool, two independent expert annotators manually curated ground‐truth mappings for each query cell type, stored as binary labels in columns manual_CL (Cell Ontology) and manual_CT (Cell Taxonomy). Predictions (predicted_CL, predicted_CT) were binarized by applying the 0.6 similarity cutoff.

We report two families of metrics.
**(i) Top-1 Exact-Match (Accuracy):** the proportion of queries whose predicted ontology ID exactly matches the curated ground-truth ID (top-1 accuracy; no similarity threshold).
**(ii) Accept/Reject @ τ=0.6 (F1 Score, Precision, Sensitivity, Specificity):** we treat *y_true*=1 if the top-1 ID is correct (per manual curation) and *y_pred*=1 if the similarity score ≥0.6 (accept). Based on the same *(y_true, y_pred)* we compute, Sensitivity (recall of the positive class), Specificity (recall of the negative class), Precision, and F1 using sklearn.metrics (v1.2.0).

# Supplementary Figures

Figure S1 | Cell-Taxonomy-based annotation of human bone-marrow single-cell transcriptomes (Zeng et al.)

(A) UMAP projection of 34,315 bone-marrow cells annotated with Cell Taxonomy (CT) identifiers at a coarse resolution. Distinct clusters correspond to early progenitors (HSC, MPP, CLP, GMP, CMP), lineage-restricted progenitors (MEP, Eo/Baso/Mast precursor, GMP-Neut, GMP-Mono) and mature immune populations including B cells, naïve and memory CD4⁺/CD8⁺ T cells, NK cells, monocytes, cDCs, pDCs and plasma cells. A small stromal fraction is tentatively labelled “Stromal cell of bone marrow”, yet the transcriptional separation from erythro-myeloid progenitors is modest, cautioning against over-interpretation of this assignment.

(B) The identical dataset re-annotated at higher resolution illustrates the hierarchical depth available in the taxonomy. Additional T-cell states emerge—central-memory, effector-memory, tissue-resident and activated subsets—together with discrete stages of B-cell development (immature B, large pre-B, early and late pro-B). Granular erythro-megakaryocytic differentiation (pro-erythroblast, orthochromatic erythroblast, megakaryocyte) and proliferative intermediates (“Cycling progenitor”, “Proliferative T”) are also resolved. Notably, several clusters assigned as “Pre-B” versus “Pro-B cycling” have overlapping marker repertoires, implying that the apparent separation may partly reflect cell-cycle effects rather than true developmental bifurcation.

Across both panels, coloured bullets denote CT IDs (CT0000xxxx), providing an explicit, machine-readable linkage to the underlying ontology. While the taxonomy offers a reproducible framework, users should scrutinise clusters with marginal transcriptional distances—especially where cell-cycle or technical variation could masquerade as biological identity.

Figure S2 | Cell-Taxonomy-based annotation of the cross-tissue human immune-cell atlas (Domínguez Conde et al.)[3]

Uniform Manifold Approximation and Projection (UMAP) of 79 k single cells profiled across 16 tissues and re-labelled with Cell Taxonomy (CT) identifiers. Sixty transcriptionally distinct clusters are recovered. Lymphoid lineages comprise (i) progressive B-cell maturation from immature-B and transitional-B through germinal-centre (GC) B and plasma cells; (ii) a continuum of T-cell states—naïve, central-memory (TCM), effector-memory (TEM), Temra, tissue-resident, follicular-helper (Tfh), regulatory (Treg), γδ-T, mucosal-associated invariant T (MAIT) and NKT cells; and (iii) three innate-lymphoid-cell branches (ILC1–3) in addition to CD56^bright and CD56^dim NK subsets. The myeloid compartment resolves classical, intermediate and non-classical monocytes, neutrophil–myeloid progenitors, conventional dendritic-cell axes (cDC1, cDC2, DC3), LAMP3^+ migratory DCs, macrophage specialisations (Kupffer, kidney-resident, alveolar) and mast cells. Haematopoietic stem and multipotent progenitors (HSC/MPP), lineage-restricted erythro-megakaryocytic (MEP) and granulocyte-monocyte (GMP) precursors, together with sporadic stromal contaminants—endothelial, epithelial and fibroblast signatures—are also detected.

Procedure

▲ CRITICAL Here we provide several functions that convert the cell names you annotated to their corresponding Cell Ontology/Taxonomy names and IDs. All analysis are performed with omicverse.single.CellOntologyMapper class.

1. The first step is to import omicverse and scanpy packages.

import scanpy as sc

```
#import pertpy as pt
import omicverse as ov
ov.plot_set()

%load_ext autoreload
%autoreload 2
```



2. Prepared datasets

import pertpy as pt

adata = pt.dt.haber_2017_regions()

adata



▲ CRITICAL STEP Before you convert you cell names, you need to annotate at first. Here, we used haber_2017_regions dataset from `pertpy` to test.

3. Download the CL Model

ov.single.download_cl(output_dir="new_ontology", filename="cl.json")



▲ CRITICAL STEP Before we start our analysis, we need to download the cl.json from Cell Ontology. But we have also provided a function named omicverse.single.download_cl() to download it automatically. The benefit of this function is that it can choose an appropriate source to download the file even if you encounter a network error.

4. Prepare the CellOntologyMapper

mapper = ov.single.CellOntologyMapper(

```
    cl_obo_file="new_ontology/cl.json",
    model_name="sentence-transformers/all-MiniLM-L6-v2",
    local_model_dir="./my_models"
)
```



▲ CRITICAL STEP Because the CellOntologyMapper rely on the NLP embedding model of SentenceTransformer. So we need to choose a NLP embedding from huggingface. Here are some recommendation. You can found the entire list from huggingface or huggingface mirror. Such as BAAI/bge-base-en-v1.5, sentence-transformers/all-MiniLM-L6-v2.

If you have calculated the embedding of cell ontology, you can load it directly.

mapper = ov.single.CellOntologyMapper(

```
    cl_obo_file="new_ontology/cl.json",
    embeddings_path='new_ontology/ontology_embeddings.pkl',
    local_model_dir="./my_models"
)
```



5. Mapping Celltype

mapping_results = mapper.map_adata(

```
    adata,
    cell_name_col='cell_label'
)
```



▲ CRITICAL STEP We can use map_adata to calculate simility between the cell name in our datasets and cell name in cell ontology.

6. Mapping Cell Types with LLM Assistance

mapper.setup_llm_expansion(

```
    api_type="openai", model='gpt-4o-2024-11-20',
    tissue_context="gut",    # tissue
    species="mouse",                   # species
   study_context="Epithelial cells from the small intestine and organoids of mice. Some of the cells were also subject to Salmonella or Heligmosomoides polygyrus infection",
    api_key="sk-*"
)
mapping_results = mapper.map_adata_with_expansion(
    adata=adata,
    cell_name_col='cell_label',
    threshold=0.5,
    expand_abbreviations=True
)
mapper.print_mapping_summary(mapping_results, top_n=15)
```



▲ CRITICAL STEP In addition, we often use abbreviations to name our cell types, such as TA and TA.Early in our data sets. Calculating similarity to the Cell Ontology directly can be confusing, so we use an LLM to expand these abbreviated cell names.

You can choose any other model api from the alternative provider, such as ohmygpt. But the format of openai should observe the rule of openai.

mapper.setup_llm_expansion(

```
    api_type="custom_openai",
    api_key="sk-*",
    model="gpt-4.1-2025-04-14",
    base_url="https://api.ohmygpt.com/v1"
)
mapping_results = mapper.map_adata_with_expansion(
    adata=adata,
    cell_name_col='cell_label',
    threshold=0.5,
    expand_abbreviations=True
)
```



7. Cell Taxonomy Mapping

To enhance the annotation of cell type, we can choose not only from Cell Ontology, we can also use Cell Taxonomy database as another reference.

wget https://download.cncb.ac.cn/celltaxonomy/Cell_Taxonomy_resource.txt -O new_ontology/Cell_Taxonomy_resource.txt



Similiarly, we can use map_adata_with_taxonomy to perform the mapping.

mapper.load_cell_taxonomy_resource("new_ontology/Cell_Taxonomy_resource.txt",

```
                                   species_filter=["Homo sapiens", "Mus musculus"])

enhanced_results = mapper.map_adata_with_taxonomy(
    adata,
    cell_name_col="cell_label",
    new_col_name="enhanced_cell_ontology",
    expand_abbreviations=True,
    use_taxonomy=True,
    species="Mus musculus",
    tissue_context="Gut",
    threshold=0.6,
)
```



8. Mapping Check

We can also query the matching result manually.

res=mapper.find_similar_cells("T helper cell", top_k=10)

```
res=mapper.find_similar_cells("Macrophage", top_k=8)

#Enterocyte.Progenitor
res=mapper.find_similar_cells_taxonomy("T helper cell", top_k=2)

mapper.get_cell_info("regulatory T cell")

#
info_list = mapper.get_cell_info_taxonomy(
    "Helper T cell",
    species="Mus musculus"
)

mapper.get_cell_info("regulatory T cells")

my_categories = ["immune cell", "epithelial"]
mapper.browse_ontology_by_category(categories=my_categories, max_per_category=5)

res=mapper.list_ontology_cells(max_display=10)
stats = mapper.get_ontology_statistics()
```



**REFERENCE**

1. Zeng, Andy G. X., Ilaria Iacobucci, Sayyam Shah, Amanda Mitchell, Gordon Wong, Suraj Bansal, David Chen, et al. 2025. “Single-cell Transcriptional Atlas of Human Hematopoiesis Reveals Genetic and Hierarchy-Based Determinants of Aberrant AML Differentiation.” *Blood Cancer Discov* 6: 307−324. <https://doi.org/10.1158/2643-3230.Bcd-24-0342>

2. Haber, Adam L., Moshe Biton, Noga Rogel, Rebecca H. Herbst, Karthik Shekhar, Christopher Smillie, Grace Burgin, et al. 2017. “A single-cell survey of the small intestinal epithelium.” *Nature* 551: 333−339. https://doi.org/10.1038/nature24489

3. Domínguez Conde, C., C. Xu, L. B. Jarvis, D. B. Rainbow, S. B. Wells, T. Gomes, S. K. Howlett, et al. 2022. “Cross-tissue immune cell analysis reveals tissue-specific features in humans.” *Science* 376: eabl5197. <https://doi.org/10.1126/science.abl5197>

4. Vieira Braga, F. A., G. Kar, M. Berg, O. A. Carpaij, K. Polanski, L. M. Simon, S. Brouwer, et al. 2019. “A cellular census of human lungs identifies novel cell states in health and in asthma.” *Nature Methods* 25: 1153−1163. <https://doi.org/10.1038/s41591-019-0468-5>

5. Arutyunyan, Anna, Kenny Roberts, KevinTroulé, Frederick C. K. Wong, Megan A. Sheridan, Ilia Kats, Luz Garcia-Alonso, et al. 2023. “Spatial multiomics map of trophoblast development in early pregnancy.” *Nature* 616: 143−151. <https://doi.org/10.1038/s41586-023-05869-0>

6. Gayoso, Adam, Romain Lopez, Galen Xing, Pierre Boyeau, Katherine Wu, Michael Jayasuriya, Edouard Melhman, Maxime Langevin, Yining Liu, Jules Samaran. 2021. “scvi-tools: a library for deep probabilistic analysis of single-cell omics data.” *bioRxiv* 2021.04.28.441833, https://doi.org/10.1101/2021.04.28.441833

7. Wang, Sheng, Angela Oliveira Pisco, Aaron McGeever, Maria Brbic, Marinka Zitnik, Spyros Darmanis, Jure Leskovec, Jim Karkanias, Russ B Altman. 2021. “Leveraging the Cell Ontology to classify unseen cell types.” *Nature Communications* 12: 5556. [https://doi.org/10.1038/s41467-021-25725-x](https://doi.org/10.1038/s41586-023-05869-0)

8. Jiang, Shuai, Qiheng Qian, Tongtong Zhu, Wenting Zong, Yunfei Shang, Tong Jin, Yuansheng Zhang, Ming Chen, Zishan Wu, Yuan Chu. 2023. “Cell Taxonomy: a curated repository of cell types with multifaceted characterization.” *Nucleic Acids Research* 51: D853−D860. https://doi.org/10.1093/nar/gkac816
